# Burden and inequalities of chronic kidney disease attributable to diet globally, regionally and temporally, 1990–2021

**DOI:** 10.3389/fnut.2025.1592389

**Published:** 2025-06-18

**Authors:** Nana Wei, Miao Yang, Pingping Zheng, Jian Xu

**Affiliations:** ^1^Department of General Medicine, The First Affiliated Hospital of Bengbu Medical University, Bengbu, Anhui, China; ^2^Department of Pharmacy, The First Affiliated Hospital of Bengbu Medical University, Bengbu, Anhui, China

**Keywords:** chronic kidney disease, diet, global burden of disease study, temporal trend, disease burden analysis

## Abstract

**Background:**

Modifiable dietary habits are a crucial means of reducing the risk of CKD. However, there is currently a lack of global-scale analysis on the burden of CKD attributable to diet. This study aimed to examine the burden of CKD potentially associated to diet globally, regionally and temporally.

**Method:**

Our research utilized data sourced from the 2021 edition of the Global Burden of Disease (GBD) study. We gathered information on the worldwide impact of diet-related CKD spanning from 1990 to 2021, categorizing this impact based on various factors including gender, age, GBD geographical regions, and individual countries. To assess the evolving trend of diet-attributable CKD burden over this period, we employed the Joinpoint regression model, calculating the average annual percent change (AAPC) for a comprehensive understanding. Cluster analysis was employed to classify countries into distinct dietary risk categories.

**Results:**

In 2021, globally, CKD burden potentially associated to diet resulted in 317,010 deaths (95% UI: 185,370–454,850) and 7,971,280 DALYs lost (95%UI: 4,630,030–11,451,430). These figures accounted for 20.75% of all CKD-related deaths and 17.93% of all CKD-related DALYs. The age-standardized mortality and DALY rates potentially associated to diet rose notably, reaching 3.83 (95%UI: 2.25–5.49) and 93.52 (95%UI: 54.29–134.38) per 100,000 population, respectively. However, significant regional variations were observed in these rates, with Central Sub-Saharan Africa experiencing the highest and Eastern Europe the lowest. High-income North America experienced a particularly steep increase, with an AAPC of 2.93% (95% CI: 2.85, 3.01%) for deaths and 2.51% (95%CI: 2.44, 2.56%) for DALYs. Among dietary factors, insufficient intake of fruits and vegetables emerged as the primary contributor to the CKD burden. By cluster analysis, seven clusters of dietary risk patterns were identified.

**Conclusion:**

Diet may play a substantial role in the burden of CKD, with notable variations across different regions. It is imperative to implement enhanced dietary guidelines, with particular attention to mitigating the challenges faced by low-income countries and reversing the upward trend in high-income countries.

## 1 Introduction

Chronic kidney disease (CKD) poses a significant challenge to global public health, with substantial impacts reported in recent studies (1). According to the latest global research, in 2021, CKD affected 8.8% of the world’s population, resulting in 1.53 million deaths and accounting for 44.45 million disability-adjusted life-years (DALYs) lost (2). Furthermore, the global burden of CKD has been increasing over the past decades, as evidenced by the rising death and DALY rates (3). Given this situation, the significance of primary prevention in addressing modifiable risk factors associated with CKD cannot be underestimated.

The link between diet and CKD is gaining significant attention, particularly as diet is a modifiable factor. Various research endeavors have highlighted a tight correlation between adjustable dietary components and both the onset and progression of CKD. For example, epidemiological studies, such as those conducted by Shivakumar et al. (4) and Kelly et al. (5), have pinpointed excessive sodium intake as a prominent risk factor for CKD. In numerous global regions, particularly densely populated Asian nations like China, sodium consumption surpasses the WHO’s recommended limits (6). Cohort studies have indicated that adhering to healthy plant-based and vegetarian diets is associated with positive outcomes in kidney disease (7). Similarly, Rebholz et al. (8) found that strict adherence to the Dietary Approaches to Stop Hypertension (DASH) diet-characterized by limited red and processed meat consumption and high intake of fruits, vegetables, and low-fat dairy-was linked to a 16% reduced risk of kidney diseases compared to those with minimal adherence. Meanwhile, mechanistic studies have suggested the potential biological plausibility underlying the diet-CKD association (9). Thus, incorporating these dietary patterns into public health initiatives could offer additional pathways for preventing CKD.

However, the methodological heterogeneity in observation periods, analytical approaches, and model configurations across existing studies poses substantial challenges to elucidating the global CKD burden that potentially associated to diet. More critically, conducting comparative assessments of CKD burden that potentially associated to diet across diverse global populations within a standardized methodological framework is essential for informing evidence-based policymaking regarding optimal food production systems and sustainable nutrition strategies for each country (10).

While previous studies have reported GBD diet-related CKD burden (11, 12), they predominantly focus on estimating attributable burdens from overall dietary patterns. This approach inadequately disentangles the independent contributions of specific dietary components. Notably, current dietary guidelines and policies target individual dietary element for precision prevention. More critically, national dietary risks frequently manifest as combinatorial effects of multiple factors, where specific dietary component interactions may exhibit latent synergistic patterns. For instance, although some studies suggest that plant-based foods have a protective effect against the development of CKD, plant-based diets may lead to deficiencies in certain nutrients and may not be suitable for all individuals (13). Therefore, comprehensive dietary intervention strategies are critical. However, existing GBD methodologies lack systematic clustering analysis based on dietary risk profile similarities.

Building upon the conventional GBD analytical framework, this study systematically quantifies the CKD-attributable burdens of seven specific dietary factors across 204 countries and territories, while implementing machine learning-driven cluster analysis to stratify nations into high-resolution subgroups based on dietary risk profiles. This dual approach identifies distinct latent classes of dietary risk patterns, thereby enabling precision-targeted multimodal dietary intervention strategies tailored to specific national contexts.

## 2 Methods

### 2.1 Overview

The 2021 GBD study conducted a comprehensive analysis of health losses linked to 369 diseases, alongside 87 risk factors, across 204 countries and territories (14). This analysis utilized the most recent epidemiological information and refined standardized approaches. Our investigation integrated data on CKD-related deaths and DALYs that potentially attributed to diet, alongside the respective age-standardized rates, considering factors like age, gender, Socio-demographic Index (SDI), and geographical location. In the GBD study, the SDI was utilized as an indicator to measure the socioeconomic standing of various nations. A higher SDI score indicates a more advanced socioeconomic condition. For the purpose of the GBD Study, countries were categorized into five groups based on their SDI scores: high, high-middle, middle, low-middle, and low. To streamline the analysis, the study grouped countries and territories with comparable traits into 21 geographical regions.

### 2.2 Estimation methods

The GBD analysis selected risk-outcome pairs by first systematically evaluating all epidemiological evidence and summarizing key relationship characteristics (effect size magnitude, dose–response relationships, biological plausibility), followed by applying World Cancer Research Fund evidence grading criteria to retain only pairs with convincing or probable evidence (14). In GBD 2021 study, seven dietary components have been pinpointed as risk factors for CKD: high consumption of processed meat, red meat, sodium, sugar-sweetened beverages, and low intake of fruits, vegetables, and whole grains. Additionally, CKD was classified under codes N18. Elsewhere (14), the methodologies employed by GBD to quantify the burden linked to dietary factors were detailed. Building upon the aforementioned methodology for establishing exposure-disease associations, the quantification of exposure levels integrates multisource data, including nationally/subnationally representative nutrition surveys employing 24-h dietary recall, food frequency questionnaires, and household budget surveys (HBS), commercial sales data from Euromonitor International and Food availability metrics from the UN Food and Agriculture Organization (FAO). Subsequently, the comparative risk assessment framework (14) was implemented to calculate population attributable fractions (PAFs) of CKD burden attributable to each dietary risk. This involved spatiotemporal Gaussian process regression for exposure surface modeling and Bayesian meta-regression (MR-BRT) to synthesize risk-outcome relationships. Moreover, a theoretical minimum risk exposure level was established to estimate the PAFs and attributable burden. By considering the number of CKD deaths and DALYs, the number of CKD deaths and DALYs attributable to dietary risks were derived. The population data from the 2021 global demographic estimates provided by GBD study were used to calculate the age-standardized rates of death and DALY.

### 2.3 Statistical analysis

The age-standardized rates for deaths and DALYs accompanied by 95% uncertainty intervals (UI) are reported for every 100,000 individuals in the population. To explore the link between the SDI and disease burden, we applied Spearman’s correlation analysis. To assess the temporal trends in the diet-related CKD burden from 1990 to 2021, we utilized a Joinpoint regression model (15). We then calculated the average annual percent change (AAPC) with its corresponding 95% confidence intervals (CI). A hierarchical clustering approach (using Ward’s method) was employed to identify exposure clusters. The optimal number of clusters was determined using the *R*^2^ statistic, defined as the ratio of the between-cluster sum of squares to the total sum of squares. The statistical analyses and visualizations were conducted using R software (version 4.2.1), with statistical significance set at a *p*-value of 0.05.

## 3 Results

### 3.1 Global burden and trend of CKD attributable to diet in 2021

In 2021, globally, the tally of deaths and DALYs of CKD potentially associated to dietary factors stood at 317,010 (with a 95% UI of 185,370–454,850) and 7,971,280 (95%UI: 4,630,030–11,451,430), respectively. These numbers represented 20.75% (95%UI: 12.20–29.38%) of all CKD-related deaths and 17.93% (95%UI: 10.51–25.31%) of all CKD DALYs. The corresponding age-standardized rates for death and DALY were 3.83 (95%UI: 2.25–5.49) and 93.52 (95%UI: 54.29–134.38) per 100,000 population in 2021. Between 1990 and 2021, these rates exhibited a significant upward trend, with an AAPC of 0.58% (95%CI: 0.55, 0.60%) for deaths and 0.36% (95%CI: 0.34, 0.38%) for DALYs, respectively.

### 3.2 Global burden of CKD attributable to diet in 2021 by gender and age

**Figure 1:**
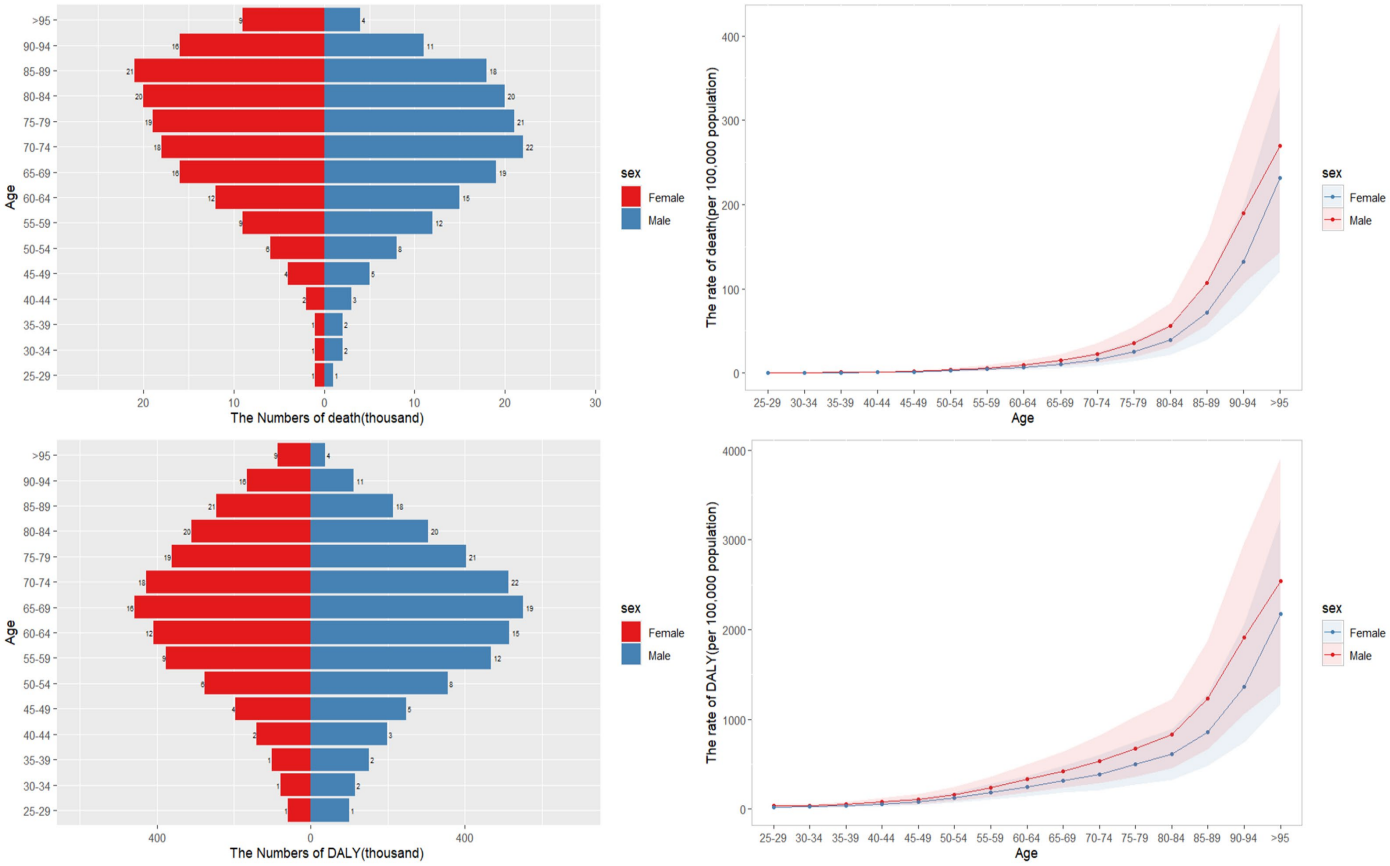
Age-specific numbers and rates of CKD death and DALY that potentially associated to diet risk by gender and age, in 2021.

In general, males exhibited higher burden of CKD potentially associated to dietary factors compared to females, with age-standardized rates of 4.56 (95%UI: 2.67–6.66) and 108.58 (95% UI: 62.74–159.15) per 100,000 population for males, and 3.28 (95% UI: 1.93–4.76) and 80.69 (95% UI: 47.55–116.70) for females, respectively. Both death and DALY rates increased with age (refer to Figure 1). Additionally, the total count of DALYs and deaths from CKD that potentially linked to diet was greater among males than females. Prior to the age group 80–84, males had a higher number of CKD deaths and DALYs that potentially attributable to diet compared to females, but this trend reversed thereafter (Figure 1).

### 3.3 The burden of CKD attributable to diet by geographical regions

**Figure 2:**
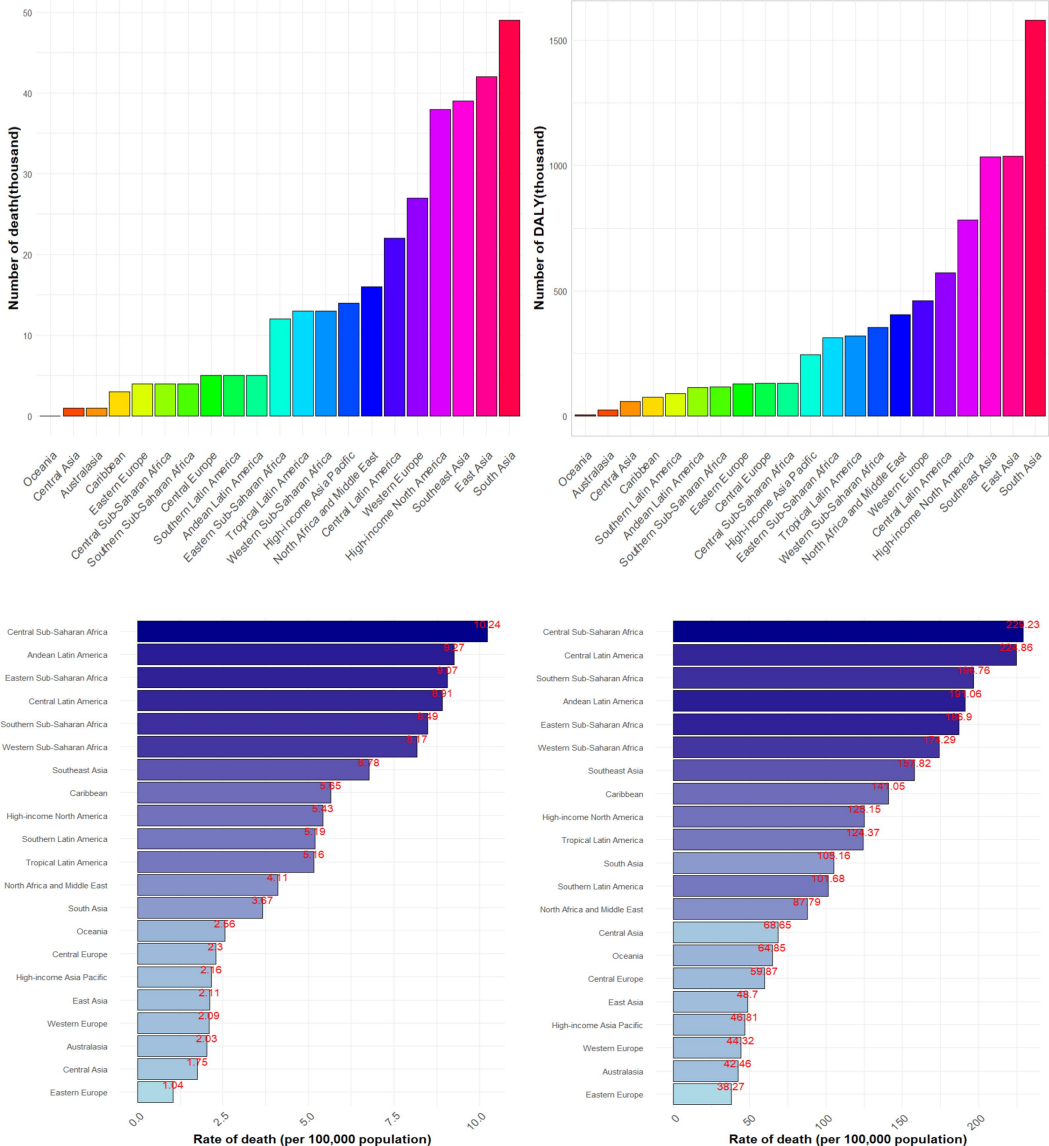
Numbers and age-standardized rates of CKD death and DALY that potentially associated to diet risk among different regions in 2021.

The graphical representation of the results is provided in Figure 2. Among the 21 regions analyzed, South Asia reported the highest number of CKD deaths (48,620, with a 95% UI of 26,200–74,990) and DALYs (15,797,100, 95% UI: 8,809,400–24,189,400) that potentially associated to dietary factors, followed by East Asia, and Southeast Asia. In terms of age-standardized rates, Central Sub-Saharan Africa ranked highest for both deaths (10.24, 95% UI: 5.54–15.96) and DALYs (229.23, 95% UI: 128.39–350.77), with Central Latin America, and Southern Sub-Saharan Africa following closely. Conversely, Eastern Europe, Australasia, and Western Europe exhibited the lowest age-standardized rates.

**Figure 3:**
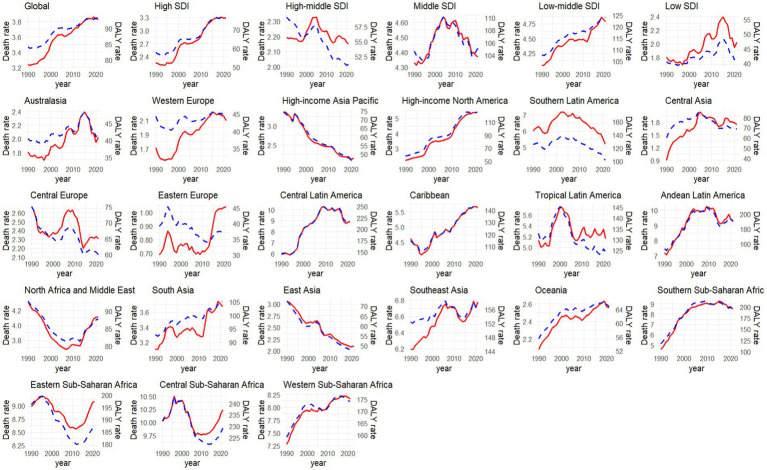
Temporal trend of age-standardized rate of CKD death and DALY that potentially associated to diet risk to diet from 1990 to 2021 among different regions (red line indicated age-standardized death rate [per 100,000 people], while blue line meant the age-standardized DALY rate [per 100,000 people]).

Figure 3 illustrates the temporal variations in the burden of CKD that potentially associated to dietary factors across diverse regions. Out of 21 regions analyzed, only five regions showed a decreasing trend in age-standardized death rates and eight regions in age-standardized DALY rates of CKD that potentially associated to diet. Notably, High-income Asia Pacific exhibited the most significant decline, with an AAPC of −1.46% (95%CI: −1.54, −1.35%) for deaths and −1.56% (95%CI: −1.64, −1.48%) for DALYs. In contrast, High-income North America stood out with the most pronounced increase, with an AAPC of 2.93% (95%CI: 2.85, 3.01%) for deaths and 2.51% (95%CI: 2.44, 2.56%) for DALY rates (Supplementary Table S1).

### 3.4 The burden of CKD attributable to diet in SDI regions

In 2021, the age-standardized death and DALY rates per 100,000 inhabitants reached their zenith in regions with low SDI, amounting to 6.69 (with a 95% UI of 3.8–10.1) and 153.1 (95%UI: 87.25–226.01), respectively. In contrast, these rates were at their lowest in High-middle SDI regions, where they stood at 2.15 (95%UI: 1.21–3.16) and 51.19 (95%UI: 29.01–75.46) (Supplementary Table S1). A notable correlation was observed between SDI and the death (*r* = −0.59, *p* < 0.001) and DALY (*r* = −0.61, *p* < 0.001) rates of CKD that potentially associated to dietary risks (Supplementary Figure S1). Between 1990 and 2021, only the High-middle SDI region exhibited a decline in the age-standardized death and DALY rates of CKD that potentially associated to dietary risks, whereas High SDI regions showed the most significant increase, with AAPC values of 1.24% (95%CI: 1.17, 1.32%) and 0.87% (95%CI: 0.83, 0.92%), respectively (Figure 3).

### 3.5 The burden of CKD attributable to diet by countries and territories

**Figure 4:**
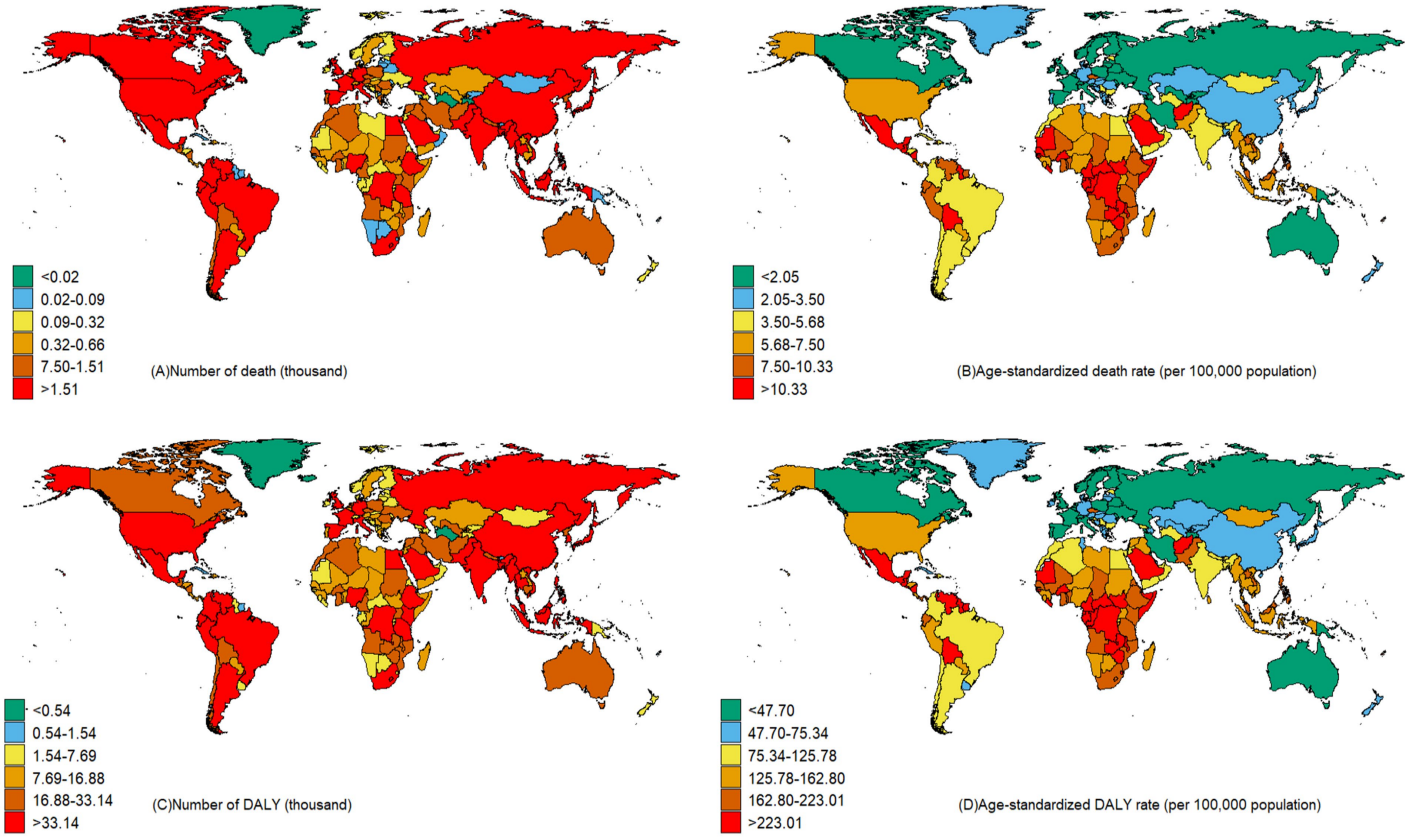
Number and age-standardized CKD death and DALY rates of CKD that potentially associated to diet risk across countries in 2021.

China and India reported the highest figures for CKD deaths and DALYs that potentially associated to diet, with 39,330 deaths (95%UI: 20,110–61,190) and 12,267,400 DALYs (95%UI: 6,752,400–18,878,900) respectively, as illustrated in Figure 4. When considering the age-standardized rate per 100,000 population for CKD that potentially associated to dietary risks, Mauritius topped the list, with a death rate of 19.90 (95%UI: 11.54–28.83) and a DALY rate of 455.36 (95%UI: 266.72–656.57), as detailed in Supplementary Table S2.

Between the years 1990 and 2021, a limited number of nations have exhibited a decrease in the CKD burden that potentially associated to diet risks. Notably, Poland has reported the greatest reduction in mortality rates (AAPC: −2.36, 95%CI: −2.50, −2.22%), while the Maldives has seen the most substantial decrease in DALY rates (AAPC: −2.57, 95%CI: −2.66, −2.46%) (Supplementary Table S2). Conversely, Ukraine has the highest rise in mortality (AAPC: 9.52, 95%CI: 8.88, 10.11%) and American Samoa reporting the largest increase in DALY rates (AAPC: 3.08, 95%CI: 3.00, 3.15%). It’s worth mentioning that the United States also demonstrates a relatively high growth rate in both mortality (AAPC: 3.07, 95%CI: 2.99, 3.15%) and DALY rates (AAPC: 2.62, 95%CI: 2.56, 2.68%) (Supplementary Table S2).

### 3.6 Diet risks subtypes and their changes for burden of CKD

**Figure 5:**
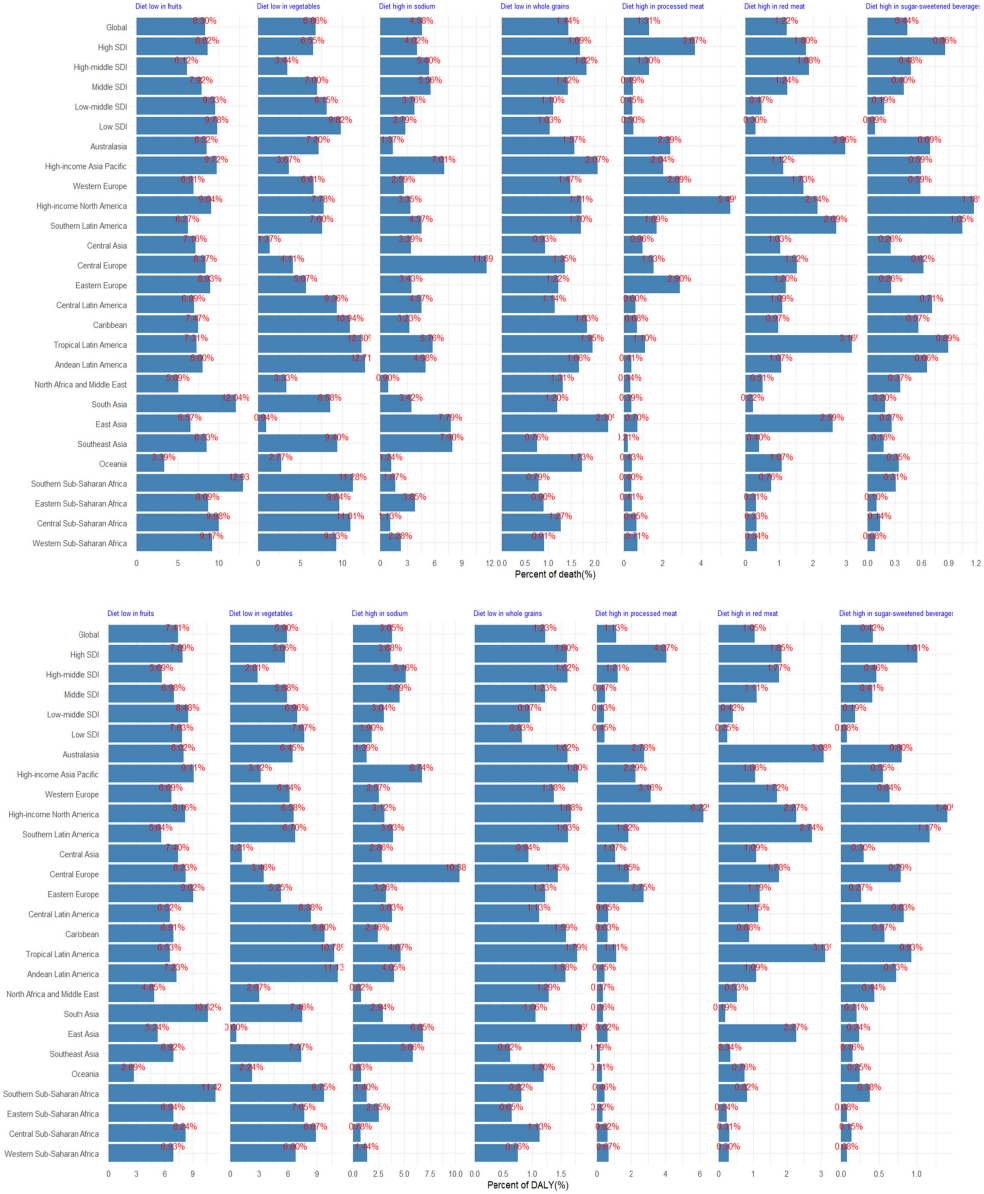
Proportion of CKD death and DALY potentially associated with specific dietary components, for global, 5 SDI, and 21 GBD regions, 2021.

In 2021, dietary factors played a substantial role in the global burden of CKD, with diets lacking in fruits and vegetables emerging as the most prominent contributors. Specifically, diets low in fruits potentially accounted for 8.30% of CKD deaths and 7.41% of DALYs, while diets low in vegetable intake was potentially responsible for 6.86% of deaths and 5.90% of DALYs (Figure 5). High-middle SDI regions faced significant challenges from high-sodium diets, whereas in high SDI regions, diets rich in processed meat posed notable risks (Figure 5). The influence of diet on the CKD burden varied across regions. Notably, high sodium intake was a major risk factor in Central Europe (accounting for 11.69% of deaths and 10.38% of DALYs), East Asia (7.79% of deaths and 6.85% of DALYs), Southeast Asia (7.90% of deaths and 5.86% of DALYs), and High-income Asia Pacific (7.01% of deaths and 6.74% of DALYs). In High-income North America, diets high in processed meat were more prevalent and contributed to a significant burden, accounting for 5.49% of deaths and 6.22% of DALYs (Figure 5). Over the past few decades, the ranking of diet contributing to CKD burden has remained consistent, with diets lacking sufficient fruit and vegetable intake consistently being at the forefront (Supplementary Figure S2).

**Figure 6:**
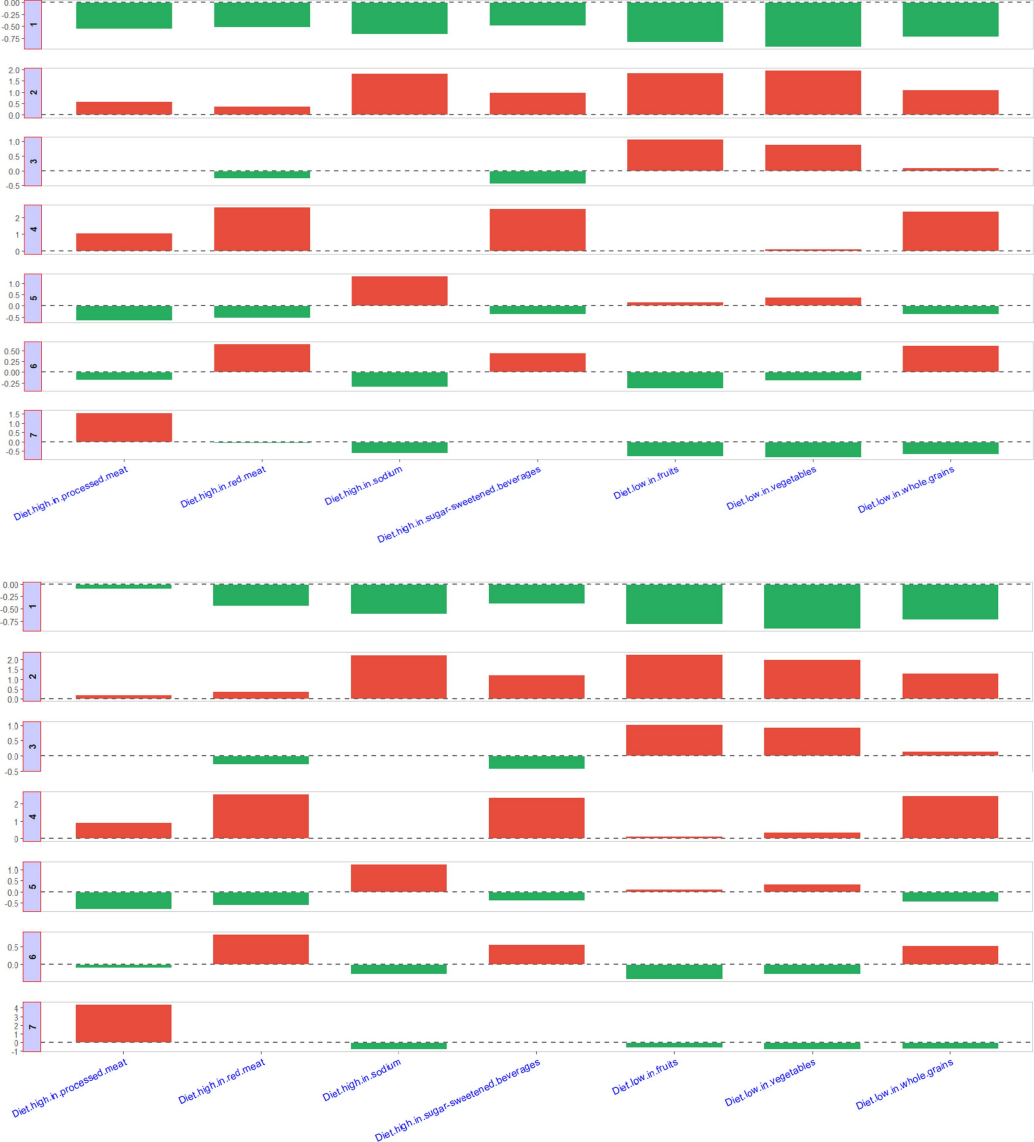
Description of seven clusters.

Figure 6 presented seven different patterns of the diet clusters. Briefly, Cluster 1 represents the most ideal state, with low diet-attributable CKD burden across all dietary factors. Cluster 2 indicates a high diet-attributable CKD burden across all dietary factors. Cluster 3 shows that the diet-attributable CKD burden is primarily driven by insufficient intake of fruits and vegetables. Cluster 4 is characterized by a diet-attributable CKD burden mainly stemming from excessive consumption of processed meat, red meat, and sugar-sweetened beverages, combined with insufficient grain intake. Cluster 5 reflects a diet-attributable CKD burden largely associated to excessive sodium intake. Cluster 6 highlights a diet-attributable CKD burden dominated by excessive intake of red meat and sugar-sweetened beverages, along with insufficient grain intake. Cluster 6 suggests a diet-attributable CKD burden primarily caused by excessive intake of processed meat. The countries or regions included in different clusters are shown in Supplementary Table S2.

## 4 Discussion

Our research provides a comprehensive analysis of the global impact of diet-related CKD from 1990 to 2021, revealing a considerable global health burden that potentially associated to dietary risks that significantly affects a large segment of the population. Notably, in 2021, diet risks contributed to a substantial proportion of CKD-related deaths (20.75%) and DALYs (17.93%), with notable variations across different regions. Moreover, the burden of CKD linked to dietary factors, both in terms of absolute numbers and age-standardized rates of death and DALYs, is showing a persistent upward trend globally. These findings hold significant implications for policymakers, particularly in the ongoing global efforts to tackle the challenges posed by CKD.

Our study suggests that the burden of CKD linked to diet is particularly high among males and older individuals, aligning with previous research findings (16, 17). This disparity could be due to males and younger adults often opting for less nutritious diets (18), combined with the delayed effects of dietary habits on health outcomes, which may increase the risk of CKD (5). Furthermore, males often lack awareness about the link between diet and overall health (19). Across all age groups, males experienced higher deaths and DALYs related to CKD compared to females, with females surpassing males only in the 80–84 age bracket. This trend may be explained by females generally having a longer lifespan and higher mortality rates among elderly populations.

Geographical variations have a profound impact on the burden of CKD linked to dietary risks across different regions. Our study found that regions such as Central Sub-Saharan Africa, Central Latin America, Southern Sub-Saharan Africa have higher burden of CKD that potentially associated to dietary factors. These areas also face significant challenges from hypertension and diabetes, which are primary risk factors for CKD (20). This disparity can be attributed to socioeconomic differences and unequal distribution of dietary habits among various regions. Our results indicate a negative correlation between SDI and the burden of CKD caused by dietary risks. High-income countries tend to promote healthy diets like the DASH and Mediterranean diets, which have been proven to lower CKD risk (21, 22). Data from 52 countries indicated that urban areas with higher incomes tend to consume more fruits and vegetables (23). Additionally, insufficient preventive care and a lack of education regarding CKD prevention and management intensify the CKD burden in these areas. Additional research is essential to uncover the root causes of regional variations in the burden of CKD linked to diet.

From 1990 to 2021, we noticed a notable increase in the burden of CKD linked to dietary risks across various regions. Notably, high-income North America, categorized under high SDI regions, exhibited the most significant surge in this trend, which mirrors the region’s CKD prevalence and mortality rates. For example, according to the GBD study, the age-standardized mortality rate for CKD rose by 57.3% in high-income North America (24). Data from the National Health and Nutrition Examination Survey (NHANES) in the United States, spanning from 1999 to 2018, revealed an escalating prevalence of early-stage CKD (25). Alarmingly, a decreasing awareness of hypertension among individuals with controlled blood pressure has emerged in recent years in the United States (26), despite hypertension being a primary risk factor for CKD (20). Furthermore, the burden of other diet-related conditions, such as stroke (27), is also on the rise in high-income North America, hinting at a potential surge in unhealthy eating habits within the region. NHANES data from 1999 to 2020 indicated that the proportion of adults consuming poor-quality diets remained high in the United States, with dietary disparities persisting or worsening (28). Our findings revealed that, compared to other regions, the consumption of processed meat contributes more significantly to diet-related CKD risk in high-income North America. Despite the International Agency for Research on Cancer (IARC) classifying processed meat as “carcinogenic to humans” and numerous epidemiological studies affirming its association with various health risks (29, 30), research suggests that the intake of processed meat in the USA has not declined over the past two decades (31).

Our investigation indicated that inadequate consumption of fruits and vegetables emerged as the leading dietary risk contributing to the CKD burden across all regions. An analysis of food surveys conducted among 143,305 individuals across 18 countries indicated that the global average daily consumption of fruits and vegetables is 3.8 servings, and 60% of the population does not meet this standard (32). Especially, residents of low-income countries consume the least number of fruits and vegetables on a daily basis (32). However, multiple studies have demonstrated a notable correlation between a diet abundant in fruits and vegetables and a lower mortality rate associated with CKD (33). These foods are typically abundant in fiber, vitamin C, potassium, carotenoids, and phenolic compounds, all of which contribute to substantially diminishing the risk of developing CKD (34). For instance, Vitamin C functions as an antioxidant, aiding in the prevention of cholesterol oxidation, inhibiting smooth muscle cell proliferation, and mitigating systemic inflammation (34). Research utilizing GBD data from both Ethiopia (35) and Australia (36) has highlighted inadequate fruit and vegetable intake as the foremost dietary factor impacting non-communicable diseases.

It is worth noting that diets rich in sodium pose a significant threat in Central Europe and Asia, particularly in China, which reports the highest deaths that potentially associated to CKD linked to dietary habits. Over the past three decades, despite a gradual reduction in sodium intake in China, the average daily salt consumption among adults still stands at 14.5 g, markedly surpassing the WHO’s recommended limit of 5 g. Excessive sodium intake is a major contributor to hypertension, a condition closely associated with CKD (37). Research from the UK Biobank indicates that individuals who occasionally, frequently, or always add salt to their meals face a 4, 7, and 11% heightened risk of developing CKD, respectively, compared to those who never or rarely do so (38). Consequently, reducing salt intake is a pivotal measure in preventing CKD in China.

Our findings should be interpreted in the context of the parallel rise in obesity and diabetes-two intersecting metabolic disorders that share common dietary drivers and may partially mediate the diet-CKD burden association (39, 40). As noted in recent studies, the global prevalence of obesity had increased by 155.1% in males and 104.9% in females between 2000 and 2021, while diabetes prevalence rose by 90.5%, creating a synergistic risk milieu for CKD progression (41, 42). Although GBD study adjusted for baseline metabolic risk factors through the comparative risk assessment framework, the dynamic interplay between temporal dietary pattern shifts and obesity/diabetes epidemics warrants further exploration. Specifically, certain dietary exposures (e.g., high sodium intake) could amplify CKD risk through both direct renal injury pathways and indirect metabolic dysregulation (43–45). This residual confounding may lead to conservative estimates of diet-attributable CKD burden in our study. Future decomposition analyses using mediation models could help disentangle the proportion of CKD burden directly attributable to dietary factors versus those mediated through obesity/diabetes pathways. Notably, future strategies for CKD prevention and control should adopt multi-pronged interventions: while promoting healthy dietary patterns, it is essential to integrate weight management and early diabetes screening, particularly in populations with higher dietary risks.

Nonetheless, we acknowledge several constraints in our research. Primarily, our conclusions might be influenced by memory bias stemming from the utilization of 24-h dietary recall methods and Food Frequency Questionnaires. Furthermore, dietary surveys were sourced from different countries, inevitably introducing variations in data quality. As a result, there may be inaccuracies in estimating the disease burdens associated with diet, either underestimated or overestimated. Secondly, the estimated effects of dietary risks on disease outcomes were primarily derived from meta-analyses of prospective observational studies. While most of these dietary relative risks were adjusted for key confounding variables (such as socioeconomic status and environmental factors), residual confounding remains a potential limitation. Thirdly, the definitions of dietary factors (e.g., whole grains) vary across studies. Moreover, since intake of healthy dietary components is generally positively correlated with each other and inversely correlated with harmful dietary components, the effect size of individual dietary factors may be overestimated. Fourthly, although GBD analytical framework determines causal relationships between specific risk factors and health outcomes through rigorous systematic evaluation of all available epidemiological evidence (14), given the inherent uncertainty in causal inference, the data presented are largely speculative. Lastly, for extensive countries like China, our analysis has not thoroughly examined such burden within their various regions.

To tackle the unequal burden of CKD linked to diet, we propose the following recommendations. Firstly, policymakers should advocate for dietary habits that cut down on sodium and red meat intake while boosting the consumption of fruits, whole grains, and vegetables. Achieving this goal can be facilitated through public health awareness initiatives, nutritional labeling, and offering tax incentives for healthier food options. Secondly, nutrition programs need to be tailored to meet the specific needs of different genders and age groups, with particular attention given to males and the elderly. Thirdly, specific actions should be undertaken in relation to the dietary habits prevalent in different countries.

## 5 Conclusion

In essence, our research revealed that inadequate diets may play a significant role in the development of CKD, and the associated dietary burden can significantly fluctuate based on yearly trends, age groups, genders, geographical locations, and SDI levels. Notably, nations with lower SDIs bore a disproportionately higher CKD burden. Considering the global aging population expansion, it is imperative to implement enhanced dietary guidelines, with particular attention to mitigating the challenges faced by low-income countries and reversing the upward trend in high-income countries.

## Data Availability

The original contributions presented in the study are included in the article/Supplementary material, further inquiries can be directed to the corresponding author.
