# Supplementary material for: Burden and inequalities of chronic kidney disease attributable to diet globally, regionally and temporally, 1990–2021
Source: Front Nutr. 2025 Jun 18;12:1592389. doi: 10.3389/fnut.2025.1592389 (PMC12213357; doi:10.3389/fnut.2025.1592389)
Supplement: Supplementary file 1 [file Table_1.docx]

**Burden and inequalities of chronic kidney disease attributable to diet globally, regionally and temporally, 1990 to 2021**

**Fig S1**. Correlation between SDI and age-standardized rates of CKD death and DALY attributable to diet in 2021

**Fig S2** Leading dietary risks of death and DALY of CKD in 19900 and 2021

**Figure S3**. Changes of R^2^ under different number of cluster (2-15) in cluster analysis

**Table S1.** Age-standardized rates of CKD death and DALY attributable to diet in different regions in 2021

**Table S2.** The temporal trend of CKD attributable to diet in 204 countries and territories, 1990-2021


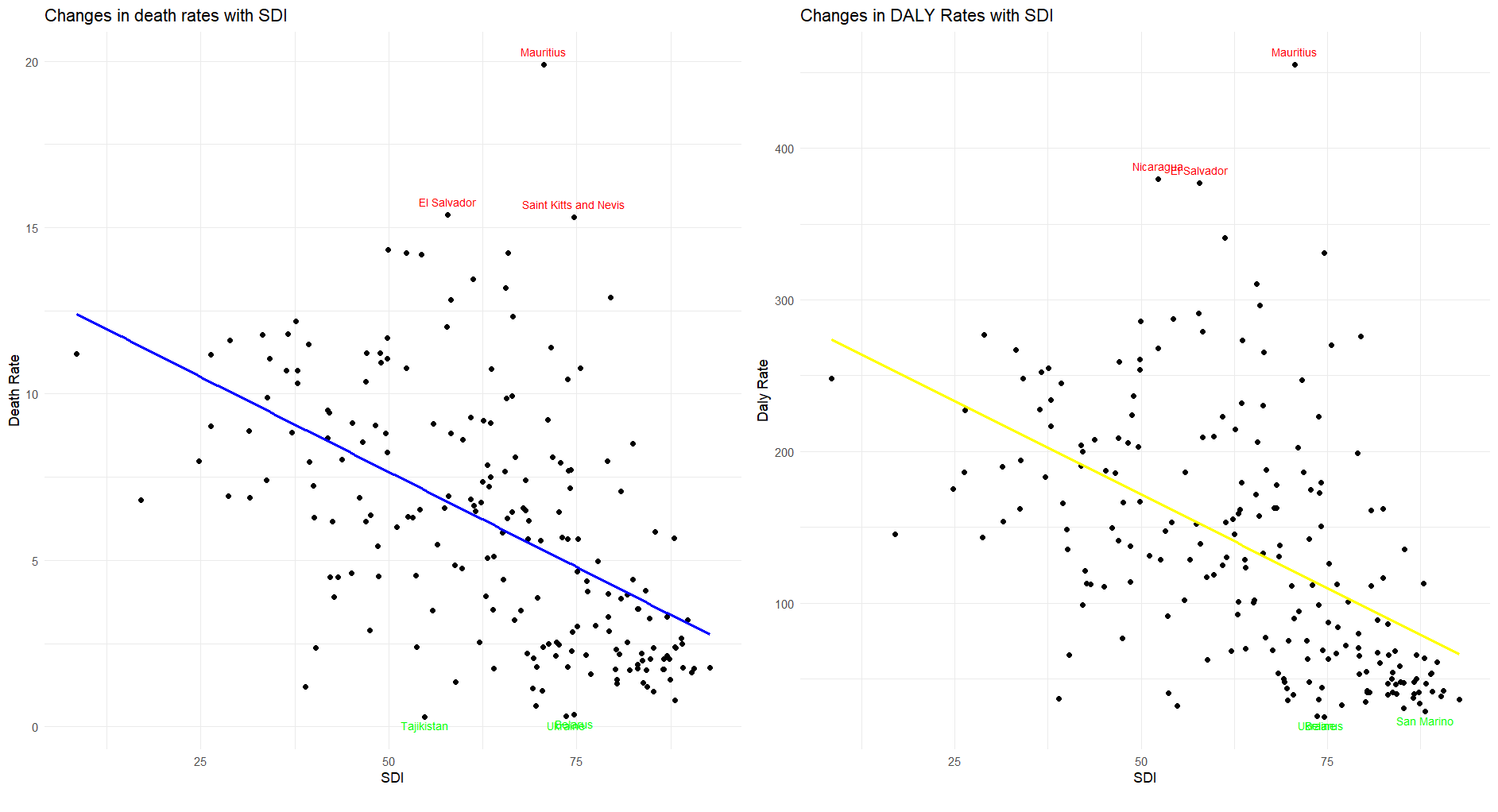


**Fig S1**. Correlation between SDI and age-standardized rates of CKD death and DALY attributable to diet in 2021

**
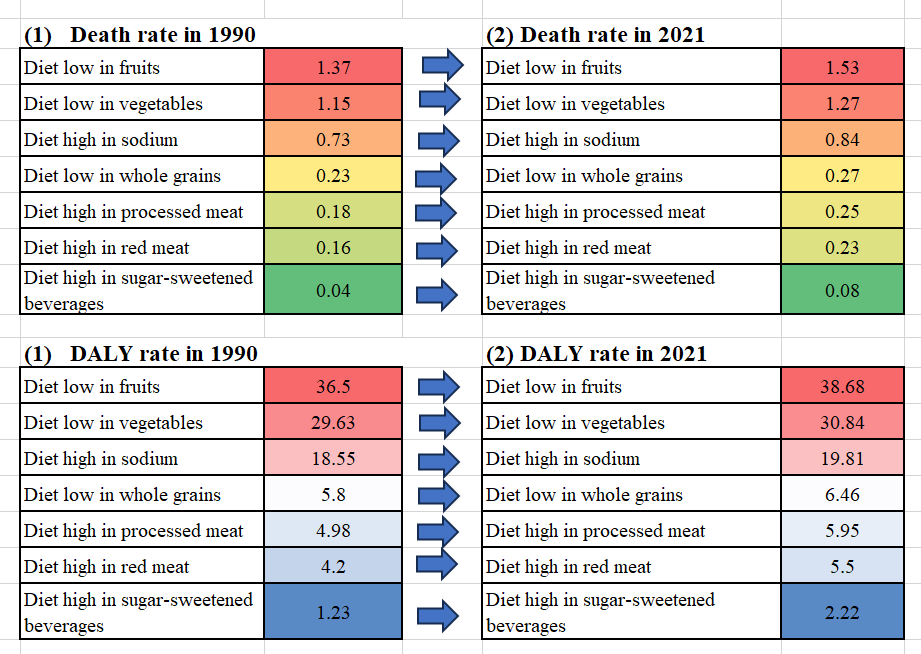
**

**Fig. S2** Leading dietary risks of death and DALY of CKD in 19900 and 2021

**
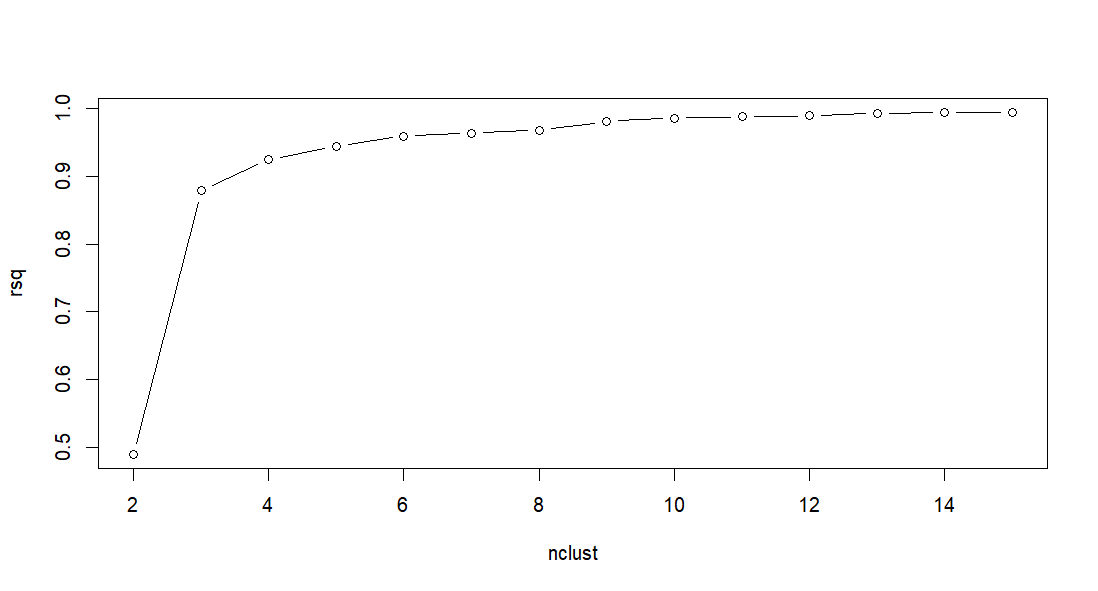
**

**
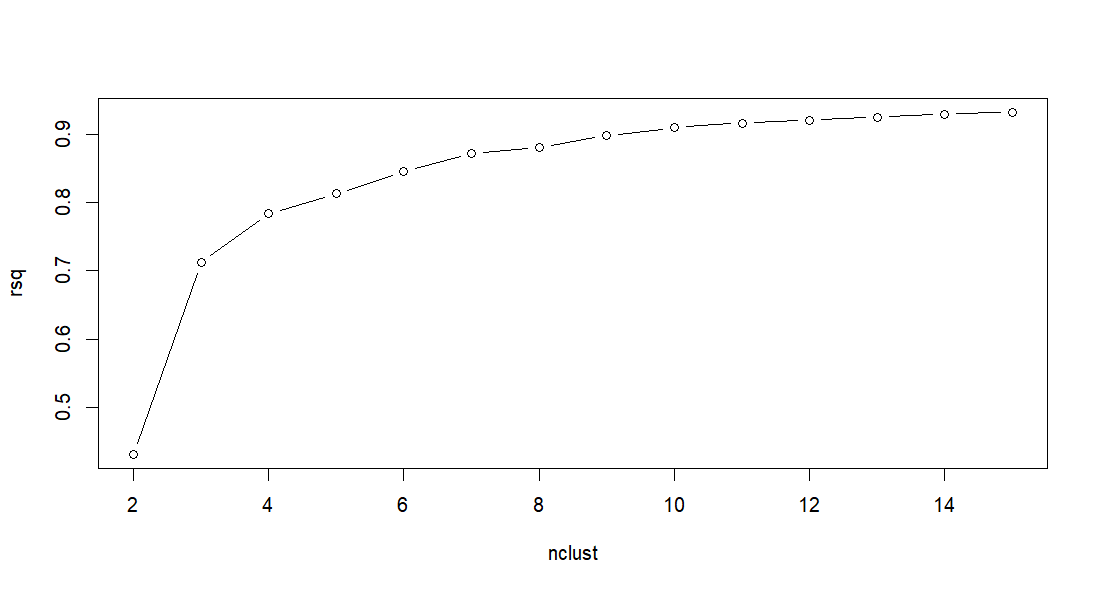
**

1. (B)

**Figure S3**. Changes of R^2^ under different number of cluster (2-15) in cluster analysis (A) Death rate (B) DALY rate

**Table S1. Age-standardized rates of CKD death and DALY attributable to diet in different regions in 2021** DALY, disability-adjusted life year; SDI, Socio-demographic Index**.**

|  | Death | | DALY | |
| --- | --- | --- | --- | --- |
|  | Age-standardized rate  (per 100000 population) | AAPC(%) | Age-standardized rate  (per 100000 population) | AAPC |
| **Global** | 3.83(2.25,5.49) | 0.57(0.55,0.60) | 93.52(54.29,134.38) | 0.36(0.34,0.38) |
| **SDI** |  |  |  |  |
| Low SDI | 6.69(3.8,10.1) | 0.12(0.09,0.14) | 153.1(87.25,226.01) | -0.03(-0.05,-0.02) |
| Low-middle SDI | 4.78(2.68,6.94) | 0.58(0.54,0.62) | 123.91(69.82,182.13) | 0.47(0.44,0.49) |
| Middle SDI | 4.37(2.56,6.38) | 0.03(-0.01,0.06) | 105.15(60.22,151.78) | 0.02(-0.03,0.05) |
| High-middle SDI | 2.15(1.21,3.16) | -0.06(-0.08,-0.04) | 51.19(29.01,75.46) | -0.46(-0.49,-0.43) |
| High SDI | 3.29(1.92,4.69) | 1.24(1.17,1.32) | 76.04(45.79,106.63) | 0.87(0.83,0.92) |
| **High income** |  |  |  |  |
| Australasia | 2.03(1.17,2.88) | 0.32(0.08,0.53) | 42.46(24.39,60.99) | 0.05(-0.13,0.25) |
| Western Europe | 2.09(1.21,3.11) | 0.71(0.65,0.79) | 44.32(25.37,64.23) | 0.02(-0.01,0.05) |
| High-income Asia Pacific | 2.16(1.17,3.32) | -1.46(-1.54,-1.35) | 46.81(25.77,70.53) | -1.56(-1.64,-1.48) |
| High-income North America | 5.43(3.29,7.44) | 2.93(2.85,3.01) | 125.15(76.36,172.48) | 2.51(2.44,2.56) |
| Southern Latin America | 5.19(2.94,7.65) | -0.48(-0.55,-0.4) | 101.68(58.55,148.07) | -0.66(-0.75,-0.57) |
| **Central Europe, eastern Europe, and central Asia** |  |  |  |  |
| Central Asia | 1.75(0.87,2.84) | 2.1(1.9,2.29) | 68.65(36.41,106.02) | 0.43(0.31,0.6) |
| Central Europe | 2.3(1.22,3.6) | -0.49(-0.56,-0.42) | 59.87(32.57,91.84) | -0.74(-0.79,-0.7) |
| Eastern Europe | 1.04(0.57,1.57) | 1.43(1.31,1.56) | 38.27(21.62,56.27) | -0.05(-0.13,0.03) |
| **Latin America and Caribbean** |  |  |  |  |
| Central Latin America | 8.91(5.06,13.03) | 1.28(1.08,1.41) | 224.86(129.21,327.66) | 1.45(1.33,1.54) |
| Caribbean | 5.65(3.38,8.11) | 0.72(0.61,0.82) | 141.05(87.18,201.24) | 0.7(0.62,0.77) |
| Tropical Latin America | 5.16(3.22,7.07) | 0.11(0.02,0.23) | 124.37(77.61,168.66) | -0.2(-0.29,-0.09) |
| Andean Latin America | 9.27(5.38,13.5) | 0.9(0.8,0.99) | 191.06(112.76,273.42) | 0.86(0.77,0.95) |
| **North Africa and Middle East** | 4.11(2.28,6.09) | -0.13(-0.16,-0.11) | 87.79(49.91,130.38) | -0.17(-0.19,-0.15) |
| **South Asia** | 3.67(1.96,5.62) | 0.61(0.55,0.67) | 105.16(58.46,160.45) | 0.45(0.4,0.51) |
| **Southeast Asia, East Asia, and Oceania** |  |  |  |  |
| East Asia | 2.11(1.08,3.28) | -1.23(-1.28,-1.18) | 48.7(25.79,74.68) | -1.29(-1.33,-1.25) |
| Southeast Asia | 6.78(3.86,9.95) | 0.3(0.28,0.33) | 157.82(88.52,235.07) | 0.12(0.1,0.14) |
| Oceania | 2.56(1.37,3.96) | 0.63(0.59,0.66) | 64.85(35.61,99.93) | 0.48(0.45,0.51) |
| **Sub-Saharan Africa** |  |  |  |  |
| Southern Sub-Saharan Africa | 8.49(4.72,12.24) | 2.02(1.92,2.1) | 196.76(110.55,283.63) | 1.68(1.6,1.78) |
| Eastern Sub-Saharan Africa | 9.07(5.08,13.9) | 0.04(0.02,0.07) | 186.9(105.39,283.6) | -0.15(-0.17,-0.14) |
| Central Sub-Saharan Africa | 10.24(5.54,15.96) | 0.06(0.04,0.08) | 229.23(128.39,350.76) | -0.04(-0.07,-0.02) |
| Western Sub-Saharan Africa | 8.17(4.44,12.31) | 0.37(0.36,0.39) | 174.29(96,261.75) | 0.28(0.27,0.3) |

**Table S2.** The temporal trend of CKD attributable to diet in 204 countries and territories, 1990-2021

|  | Death | |  | DALY | |  |
| --- | --- | --- | --- | --- | --- | --- |
|  | Age-standardized rate (per 100000 people) in 2021 | AAPC (%) | Cluster | Age-standardized rate (per 100000 people) in 2021 | AAPC (%) | Cluster |
| **Central Asia** |  |  |  |  |  |  |
| Armenia | 1.08(0.44,2.05) | 6.93(6.57,7.3) | 1 | 39.66(17.89,70.6) | 1.17(1.01,1.33) | 1 |
| Azerbaijan | 1.16(0.51,2.08) | 0.62(0.55,0.69) | 1 | 50.22(25.56,81.34) | -0.36(-0.4,-0.31) | 1 |
| Georgia | 2.39(1.09,3.81) | 4.62(3.97,5.23) | 1 | 89.83(45.85,136.88) | 1.91(1.59,2.22) | 1 |
| Kazakhstan | 2.14(1.03,3.59) | 2.12(1.72,2.51) | 1 | 75.17(39.13,117.9) | -0.01(-0.14,0.16) | 1 |
| Kyrgyzstan | 1.35(0.66,2.2) | 2.28(2.02,2.56) | 1 | 62.82(34.65,95.21) | 0.19(0.05,0.36) | 1 |
| Mongolia | 3.53(1.86,5.46) | -0.58(-0.69,-0.44) | 1 | 128.41(69.95,189.16) | -0.66(-0.73,-0.57) | 6 |
| Tajikistan | 0.29(0.11,0.56) | 0.78(0.71,0.86) | 1 | 32(16.15,50.74) | -0.04(-0.05,-0.02) | 1 |
| Turkmenistan | 2.49(1.22,4.42) | 1.52(1.29,1.75) | 1 | 94.82(49.48,161.69) | 0.53(0.39,0.67) | 6 |
| Uzbekistan | 1.75(0.83,3.03) | 2.67(2.19,3.2) | 1 | 70.17(36.23,115.21) | 1.05(0.76,1.33) | 1 |
| Eastern Europe |  |  |  |  |  |  |
| Belarus | 0.36(0.2,0.56) | 2.77(2.35,3.12) | 1 | 24.9(13.85,37.81) | 0.37(0.26,0.47) | 1 |
| Estonia | 3.54(2.12,5.02) | 3.78(3.37,4.19) | 7 | 85.96(49.53,124.01) | 1.61(1.45,1.76) | 7 |
| Latvia | 1.71(0.91,2.61) | 3.79(3.61,3.97) | 7 | 60.34(33.86,90.36) | 1.5(1.42,1.57) | 7 |
| Lithuania | 1.32(0.72,2.03) | 3.37(3.14,3.58) | 7 | 54.19(30.14,82.91) | 1.29(1.18,1.41) | 7 |
| Republic of Moldova | 0.64(0.34,0.98) | 2.51(2.11,2.93) | 1 | 43.76(23.78,64.73) | 1.12(1.01,1.23) | 1 |
| Russian Federation | 1.29(0.7,1.95) | 0.88(0.75,1.00) | 1 | 41.96(23.35,61.82) | -0.49(-0.58,-0.41) | 1 |
| Ukraine | 0.32(0.16,0.54) | 9.52(8.88,10.11) | 1 | 25.34(13.82,39.92) | 1.51(1.39,1.63) | 1 |
| North Africa and Middle East |  |  |  |  |  |  |
| Afghanistan | 11.79(5.49,21.97) | 0.39(0.37,0.41) | 3 | 266.89(122.52,497.1) | 0.23(0.21,0.27) | 3 |
| Algeria | 5.83(3.13,8.94) | 0.22(0.16,0.26) | 6 | 100.5(55.25,150.84) | -0.17(-0.2,-0.14) | 1 |
| Bahrain | 5.65(3.1,8.85) | 0.51(0.34,0.73) | 6 | 98.62(55.23,154.61) | 0.14(0.06,0.22) | 6 |
| Egypt | 5.07(2.53,8.51) | -0.68(-0.81,-0.55) | 6 | 101.03(52.11,171.62) | -0.49(-0.59,-0.39) | 6 |
| Iran | 1.8(0.92,2.73) | -1.09(-1.16,-1.02) | 1 | 36.15(19.48,55.32) | -1.33(-1.4,-1.27) | 1 |
| Iraq | 6.46(3.32,10.38) | 0.73(0.65,0.8) | 3 | 132.88(68.8,216.68) | 0.31(0.25,0.36) | 3 |
| Jordan | 5.68(3.16,8.48) | 0.18(-0.02,0.36) | 6 | 111.91(64.26,164.91) | -0.06(-0.17,0.06) | 6 |
| Kuwait | 2.36(1.2,3.84) | -1.53(-1.98,-1.22) | 1 | 47.67(24.47,76.08) | -1.86(-2.09,-1.64) | 1 |
| Lebanon | 2.47(1.28,4.05) | 1.11(1.07,1.15) | 1 | 48.14(26.14,78.36) | 0.89(0.81,0.95) | 1 |
| Libya | 7.16(3.6,11.48) | 2.19(2.08,2.3) | 3 | 150.57(79.34,240.47) | 2.09(2,2.17) | 3 |
| Morocco | 4.54(2.34,7.02) | 0.08(0.06,0.11) | 1 | 91.3(47.97,139.52) | -0.27(-0.3,-0.25) | 1 |
| Palestine | 6.83(3.63,10.24) | -1(-1.05,-0.95) | 3 | 124.87(69.58,188.68) | -1.19(-1.24,-1.14) | 1 |
| Oman | 4.96(2.77,7.72) | 1.61(1.5,1.71) | 6 | 100.82(57.68,154.57) | 1.27(1.21,1.32) | 6 |
| Qatar | 3.55(1.68,5.83) | 0.03(-0.36,0.35) | 4 | 66.01(30.68,108.47) | 0.12(-0.09,0.32) | 6 |
| Saudi Arabia | 12.91(7.56,19.76) | 2.29(2.25,2.32) | 4 | 275.97(159.28,426.24) | 2.22(2.19,2.25) | 4 |
| Sudan | 6.01(3.28,9.7) | 0.93(0.9,0.95) | 1 | 131.34(70.34,210.22) | 0.69(0.66,0.71) | 3 |
| Syrian Arab Republic | 7.36(3.75,12.09) | 1.14(1.04,1.23) | 3 | 145.45(78.54,239.01) | 0.96(0.9,1.02) | 3 |
| Tunisia | 3.5(1.84,5.71) | 0.09(0.03,0.15) | 1 | 68.72(36.71,110.03) | -0.15(-0.22,-0.08) | 1 |
| Turkey | 1.79(0.91,3.16) | -1.18(-1.3,-1.07) | 1 | 36.43(18.33,60.66) | -1.27(-1.33,-1.2) | 1 |
| United Arab Emirates | 5.67(2.74,8.96) | 3.34(2.59,3.96) | 6 | 113.04(60,173.75) | 2.59(2.33,2.82) | 6 |
| Yemen | 4.49(2.23,7.49) | 0.2(0.17,0.22) | 1 | 98.61(50.45,161.54) | 0(-0.04,0.03) | 1 |
| East Asia |  |  |  |  |  |  |
| China | 2.06(1.05,3.21) | -1.26(-1.3,-1.21) | 1 | 47.98(25.31,73.67) | -1.32(-1.37,-1.28) | 1 |
| Democratic People's Republic of Korea | 2.4(1.3,3.85) | -0.17(-0.18,-0.16) | 1 | 63.6(35.31,98.49) | -0.1(-0.11,-0.09) | 1 |
| Taiwan (Province of China) | 3.31(1.68,5.09) | -1.44(-1.6,-1.28) | 6 | 65.57(32.93,99.88) | -1.35(-1.45,-1.25) | 6 |
| Central Europe |  |  |  |  |  |  |
| Albania | 2.21(1,4.04) | -1.38(-1.52,-1.26) | 7 | 53.88(25.46,93.15) | -1.61(-1.69,-1.53) | 1 |
| Bosnia and Herzegovina | 2.53(1.2,4.25) | -0.05(-0.15,0.06) | 1 | 63.23(31.66,101.69) | -0.56(-0.66,-0.43) | 1 |
| Bulgaria | 4.37(2.27,6.84) | 2.46(2.2,2.75) | 7 | 112.59(58.71,174.63) | 1.9(1.71,2.19) | 6 |
| Croatia | 3.31(1.6,5.42) | 0.91(0.73,1.1) | 1 | 70.34(33.96,111.88) | 0.21(0.09,0.33) | 1 |
| Czech Republic | 1.74(0.9,2.78) | -0.72(-0.84,-0.59) | 1 | 46.87(24.85,72.32) | -1.25(-1.33,-1.15) | 1 |
| Hungary | 2.88(1.5,4.56) | 1.53(1.37,1.69) | 1 | 65.23(35.16,102.01) | 0.25(0.16,0.34) | 1 |
| North Macedonia | 2.85(1.3,5.06) | 0.65(0.55,0.74) | 1 | 68.89(32.47,116.89) | -0.01(-0.06,0.04) | 1 |
| Montenegro | 4.01(1.83,7.33) | 0.87(0.74,0.99) | 5 | 79.92(37.19,139.55) | 0.16(0.08,0.26) | 1 |
| Poland | 1.42(0.75,2.21) | -2.36(-2.5,-2.22) | 1 | 40.89(21.06,62.29) | -2.09(-2.18,-2.01) | 1 |
| Romania | 2.14(1.06,3.5) | -0.68(-0.89,-0.46) | 1 | 66.79(34.86,103.85) | -0.63(-0.77,-0.51) | 1 |
| Serbia | 4.06(2.12,6.61) | -0.43(-0.52,-0.31) | 5 | 84.13(43.87,133.67) | -0.56(-0.64,-0.47) | 5 |
| Slovakia | 2.54(1.39,4.02) | -0.74(-0.8,-0.69) | 1 | 67.36(37.12,103.14) | -0.93(-0.98,-0.87) | 1 |
| Slovenia | 1.7(0.85,2.78) | 0.11(-0.13,0.34) | 1 | 46.38(24.35,71.97) | -0.54(-0.71,-0.38) | 1 |
| Southern Sub-Saharan Africa |  |  |  |  |  |  |
| Botswana | 7.21(3.91,11.48) | 0.66(0.56,0.75) | 3 | 161.5(91.6,255.2) | 0.43(0.35,0.5) | 3 |
| Lesotho | 11.06(5.88,17.6) | 2.9(2.78,3.02) | 3 | 260.97(137.15,419.05) | 2.89(2.8,2.97) | 3 |
| Namibia | 6.65(3.52,10.21) | 0.52(0.46,0.58) | 3 | 153.55(82.86,233.91) | 0.35(0.28,0.4) | 3 |
| South Africa | 8.11(4.5,11.77) | 2.23(2.11,2.33) | 3 | 187.67(105.4,270.94) | 1.72(1.62,1.84) | 3 |
| Eswatini | 12.03(6.51,19.31) | 1.09(1.01,1.16) | 3 | 291.23(152.69,483.05) | 1.28(1.23,1.33) | 3 |
| Zimbabwe | 11.24(5.96,17.55) | 1.55(1.47,1.63) | 3 | 259.38(134.95,413.54) | 1.73(1.66,1.81) | 3 |
| Southern Latin America |  |  |  |  |  |  |
| Argentina | 5.58(3.18,8.12) | -0.65(-0.81,-0.51) | 4 | 111.12(63.68,160.94) | -0.78(-0.91,-0.66) | 6 |
| Chile | 4.67(2.66,7.16) | 0.35(0.14,0.53) | 7 | 87.07(49.8,131.2) | -0.15(-0.31,-0.01) | 1 |
| Uruguay | 3.88(2.19,5.74) | 0.23(0.03,0.41) | 7 | 75.34(41.71,110.89) | 0.04(-0.15,0.21) | 1 |
| High-income North America |  |  |  |  |  |  |
| Canada | 2.05(1.15,3.02) | 0.71(0.59,0.82) | 1 | 40.34(23.71,58.4) | 0.57(0.49,0.65) | 1 |
| Greenland | 3.01(1.62,4.54) | 0.01(-0.09,0.13) | 7 | 62.99(35.33,94.39) | -0.03(-0.1,0.06) | 1 |
| United States of America | 5.85(3.54,7.98) | 3.07(2.99,3.15) | 7 | 135.45(83,187.12) | 2.62(2.56,2.68) | 7 |
| Oceania |  |  |  |  |  |  |
| American Samoa | 9.21(4.03,15.52) | 3.45(3.35,3.54) | 4 | 202.62(90.45,345.37) | 3.08(3,3.16) | 4 |
| Micronesia (Federated States of) | 6.56(3,11.38) | 1.43(1.4,1.47) | 4 | 152.45(75.79,258.51) | 1.29(1.26,1.32) | 4 |
| Fiji | 7.67(3.61,12.48) | 2.35(2.28,2.43) | 4 | 171.55(81.96,282.98) | 1.82(1.76,1.9) | 4 |
| Guam | 3.85(2.26,5.75) | -0.88(-1.17,-0.61) | 6 | 111.35(65.35,167.78) | 0.13(0,0.26) | 6 |
| Kiribati | 4.61(2.04,8.14) | 1.17(1.14,1.19) | 6 | 110.62(52.87,190.29) | 0.88(0.87,0.9) | 6 |
| Marshall Islands | 6.53(1.49,18.36) | 2.17(2.09,2.23) | 4 | 153.53(43.56,394.75) | 1.87(1.82,1.92) | 4 |
| Cook Islands | 3.03(1.5,4.84) | 1.17(1.09,1.23) | 6 | 72.28(37.56,114.68) | 0.91(0.86,0.95) | 6 |
| Northern Mariana Islands | 5.65(2.52,9.31) | 0.41(0.07,0.64) | 4 | 125.78(60.16,204.07) | 0.47(0.38,0.55) | 4 |
| Nauru | 7.5(3.59,14.44) | 1.64(1.6,1.66) | 4 | 179.27(88.99,327.6) | 1.51(1.48,1.54) | 4 |
| Niue | 7.93(3.05,15.51) | 2.33(2.29,2.37) | 4 | 174.81(71.38,338.94) | 1.96(1.92,1.99) | 4 |
| Palau | 6.45(3.03,10.7) | 1.90(1.85,1.95) | 4 | 142.12(68.63,228.82) | 1.53(1.49,1.56) | 4 |
| Papua New Guinea | 1.21(0.66,2) | 0.19(0.13,0.23) | 1 | 36.78(21.44,56.75) | 0.1(0.08,0.13) | 1 |
| Samoa | 5.48(2.76,8.62) | 1.46(1.42,1.49) | 4 | 128.75(67.12,207.22) | 1.28(1.25,1.31) | 4 |
| Solomon Islands | 2.38(1.26,3.79) | -0.59(-0.70,-0.47) | 1 | 65.68(37.6,102.68) | -0.49(-0.58,-0.39) | 1 |
| Tokelau | 4.43(2.18,7.74) | 1.61(1.59,1.63) | 6 | 102.02(54.35,168.37) | 1.28(1.27,1.3) | 6 |
| Tonga | 2.55(1.25,4.29) | 1.72(1.67,1.78) | 1 | 68.29(34.75,114.11) | 1.22(1.18,1.27) | 1 |
| Tuvalu | 4.86(2.36,8.2) | 1.33(1.31,1.34) | 6 | 117.21(62.7,196.1) | 1.07(1.05,1.08) | 6 |
| Vanuatu | 4.52(2.21,8.19) | 0.8(0.78,0.83) | 6 | 114.23(59.05,206.03) | 0.79(0.76,0.82) | 6 |
| South Asia |  |  |  |  |  |  |
| Bangladesh | 2.89(1.55,4.47) | 0.06(-0.06,0.18) | 1 | 76.53(41.25,117.89) | -0.25(-0.31,-0.18) | 1 |
| Bhutan | 4.5(2.35,7.41) | 0.5(0.47,0.53) | 1 | 112.2(59.65,177.22) | 0.13(0.1,0.16) | 1 |
| India | 3.5(1.88,5.38) | 0.66(0.58,0.74) | 1 | 101.71(55.62,155.58) | 0.44(0.36,0.53) | 1 |
| Nepal | 3.91(2.17,6.25) | 0.88(0.84,0.91) | 1 | 113.02(64.68,173.28) | 0.4(0.36,0.43) | 1 |
| Pakistan | 6.36(3.37,9.71) | 1.15(1.13,1.2) | 3 | 166.44(90.55,251.59) | 1.12(1.09,1.15) | 3 |
| Central Latin America |  |  |  |  |  |  |
| Colombia | 3.91(2.08,5.99) | -0.71(-0.92,-0.49) | 1 | 92.54(48.91,141.17) | -0.75(-0.94,-0.55) | 5 |
| Costa Rica | 6.57(3.58,10.08) | 1.77(1.51,2.06) | 5 | 162.78(88.13,246.94) | 1.39(1.18,1.61) | 5 |
| El Salvador | 15.38(8.21,25.06) | 3.19(3.00,3.36) | 2 | 377.06(198.88,612.51) | 3(2.82,3.18) | 2 |
| Guatemala | 10.76(5.91,16.63) | 1.3(1.09,1.58) | 5 | 268.12(144.75,414.71) | 1.69(1.5,1.92) | 2 |
| Honduras | 5.44(2.82,8.71) | 2.32(2.23,2.4) | 5 | 137.71(72.7,215.72) | 1.62(1.56,1.68) | 5 |
| Mexico | 10.76(6.09,15.76) | 1.13(0.97,1.26) | 4 | 273.2(158.47,401.65) | 1.53(1.33,1.68) | 4 |
| Nicaragua | 14.24(7.61,22.21) | 2.24(2.07,2.38) | 2 | 379.74(200.66,595.5) | 2.16(1.97,2.32) | 2 |
| Panama | 7.41(4.09,11.74) | 2.73(2.52,2.93) | 5 | 177.77(100.32,275.47) | 2.09(1.91,2.25) | 5 |
| Venezuela (Bolivarian Republic of) | 9.29(4.85,15.06) | 2.76(2.59,2.91) | 5 | 223.12(119.26,357.41) | 2.26(2.09,2.42) |  |
| Eastern Sub-Saharan Africa |  |  |  |  |  |  |
| Burundi | 6.93(3.44,11.9) | -0.32(-0.34,-0.3) | 5 | 143.15(71.27,238.38) | -0.55(-0.57,-0.53) | 5 |
| Comoros | 9.12(4.68,15.21) | 0.46(0.42,0.5) | 5 | 187.36(95.12,312.24) | 0.29(0.22,0.36) | 5 |
| Djibouti | 10.38(5.41,17.73) | 1.28(1.25,1.3) | 3 | 209.02(110.44,355.05) | 1.06(1.03,1.09) | 3 |
| Eritrea | 7.95(4.02,14.26) | 0.63(0.6,0.65) | 5 | 165.93(80.78,295.05) | 0.27(0.24,0.3) | 5 |
| Ethiopia | 9.89(5.65,15.23) | -1.2(-1.23,-1.18) | 3 | 194.25(112.42,302.35) | -1.46(-1.48,-1.44) | 3 |
| Kenya | 8.24(4.5,12.73) | 1.25(1.22,1.28) | 3 | 167.02(92.17,257.91) | 1.15(1.13,1.17) | 3 |
| Madagascar | 6.3(3.23,10.2) | 0.3(0.25,0.35) | 5 | 135.54(71.07,219.37) | 0.22(0.17,0.28) | 5 |
| Malawi | 10.71(5.7,17.2) | 0.59(0.55,0.63) | 5 | 227.89(120.6,362.23) | 0.47(0.44,0.5) | 5 |
| Mozambique | 8.89(4.54,14.82) | 1.14(1.1,1.17) | 5 | 189.73(93.56,317.58) | 1.06(1.04,1.09) | 5 |
| Rwanda | 6.18(2.94,10.6) | -0.73(-0.77,-0.69) | 5 | 121.38(59.53,205.76) | -1.09(-1.13,-1.05) | 5 |
| Somalia | 11.21(5.79,19) | 0.36(0.34,0.37) | 3 | 248.27(127.87,426.47) | 0.35(0.34,0.37) | 3 |
| South Sudan | 12.18(6.17,20.18) | 1.19(1.16,1.22) | 2 | 254.72(131.7,427.17) | 1.14(1.1,1.17) | 3 |
| United Republic of Tanzania | 9.5(4.91,15.09) | 0.42(0.4,0.45) | 5 | 203.95(106.69,313.97) | 0.35(0.33,0.37) | 5 |
| Uganda | 7.24(3.58,12.62) | 0.6(0.58,0.63) | 5 | 148.33(72.61,253.13) | 0.48(0.46,0.51) | 5 |
| Zambia | 11.67(6.33,19.31) | 0.55(0.49,0.6) | 3 | 253.7(133.89,422.43) | 0.64(0.6,0.67) | 3 |
| Western Europe |  |  |  |  |  |  |
| Andorra | 1.78(0.94,2.74) | -1.19(-1.35,-1.03) | 7 | 41.61(22.79,61.48) | -0.89(-1,-0.8) | 1 |
| Austria | 3.26(1.78,4.89) | 2.8(2.61,2.97) | 7 | 58.55(33.6,86.6) | 1.27(1.18,1.35) | 1 |
| Belgium | 2.05(1.11,3.15) | 0.04(-0.08,0.15) | 7 | 47.7(27.09,71.59) | -0.31(-0.37,-0.22) | 1 |
| Cyprus | 4.1(2.24,6.19) | -1.95(-2.08,-1.82) | 7 | 68.25(38.03,100.54) | -1.58(-1.66,-1.49) | 1 |
| Denmark | 2.65(1.53,3.97) | 2.66(2.5,2.8) | 7 | 52.99(30.63,79.47) | 1.06(0.94,1.23) | 1 |
| Finland | 1.06(0.52,1.6) | 0.98(0.8,1.12) | 1 | 30.73(16.81,46.2) | -0.09(-0.16,0) | 1 |
| France | 1.87(1.11,2.7) | -0.05(-0.14,0.09) | 1 | 39.8(23.78,57.81) | 0.03(-0.06,0.1) | 1 |
| Germany | 3.2(1.7,4.82) | 1.7(1.56,1.89) | 7 | 60.89(33.92,89.4) | 0.4(0.31,0.53) | 1 |
| Monaco | 1.63(0.74,2.68) | 1.13(1.1,1.17) | 7 | 38.55(18.2,64.17) | 0.55(0.52,0.57) | 1 |
| Greece | 2.87(1.44,4.62) | 0.17(-0.2,0.52) | 7 | 53.37(26.29,86.14) | 0.34(0.11,0.56) | 1 |
| Iceland | 1.41(0.78,2.05) | 1.17(1,1.35) | 1 | 33.69(19.19,49.28) | 0.44(0.34,0.54) | 1 |
| Ireland | 1.73(0.98,2.56) | -0.33(-0.5,-0.15) | 1 | 48.01(28.02,70.81) | -0.63(-0.76,-0.44) | 1 |
| Israel | 2.17(1.06,3.68) | -1.09(-1.35,-0.78) | 1 | 40.96(20.3,70.17) | -0.89(-1.1,-0.66) | 1 |
| Italy | 1.74(0.92,2.62) | 0.1(0.03,0.17) | 1 | 35.08(18.94,52.16) | -0.47(-0.51,-0.44) | 1 |
| Luxembourg | 2.49(1.33,3.8) | 0.04(-0.12,0.21) | 7 | 53.91(31.1,78.94) | -0.36(-0.46,-0.26) | 1 |
| Malta | 2.32(1.22,3.54) | -0.7(-0.83,-0.57) | 7 | 54.61(29.34,83.28) | -0.6(-0.7,-0.49) | 1 |
| Netherlands | 2.38(1.38,3.45) | 1.77(1.61,1.98) | 1 | 46.95(27.16,67.76) | 0.81(0.71,0.94) | 1 |
| Norway | 1.75(1.01,2.5) | 1.82(1.68,1.97) | 7 | 42.33(24.67,60.8) | 0.46(0.31,0.59) | 1 |
| San Marino | 0.8(0.38,1.29) | -1.46(-1.77,-1.16) | 1 | 28.4(15.88,43.76) | -0.61(-0.74,-0.48) | 1 |
| Portugal | 2.27(1.24,3.47) | 0.01(-0.08,0.1) | 1 | 44.04(23.93,65.84) | -0.42(-0.48,-0.37) | 1 |
| Spain | 1.57(0.88,2.37) | -0.87(-0.94,-0.81) | 1 | 32.85(18.51,48.31) | -0.91(-0.96,-0.86) | 1 |
| Sweden | 2.04(1.12,3.05) | 2.53(2.34,2.66) | 7 | 41.17(23.24,61.22) | 0.89(0.8,0.97) | 1 |
| Switzerland | 1.77(0.99,2.69) | 0.62(0.47,0.76) | 1 | 36.17(20.76,53.63) | -0.17(-0.26,-0.1) | 1 |
| United Kingdom | 1.2(0.66,1.8) | 0.34(0.22,0.48) | 1 | 40.14(22.96,58.7) | -0.16(-0.22,-0.1) | 1 |
| Andean Latin America |  |  |  |  |  |  |
| Bolivia (Plurinational State of) | 14.2(8.03,21.53) | 0.97(0.92,1.02) | 2 | 287.63(164.55,439.54) | 0.78(0.73,0.83) | 2 |
| Ecuador | 9.87(5.67,15.12) | 1.76(1.56,1.92) | 4 | 206(115.34,332.32) | 1.65(1.37,1.88) | 4 |
| Peru | 7.87(4.26,11.79) | 0.46(0.19,0.74) | 5 | 159.12(87.49,239.05) | 0.3(0.05,0.53) | 5 |
| Central Sub-Saharan Africa |  |  |  |  |  |  |
| Angola | 8.55(4.63,13.69) | -0.07(-0.12,-0.02) | 3 | 185.76(98.44,295.25) | -0.34(-0.38,-0.3) | 3 |
| Central African Republic | 11.61(5.92,18.72) | 0.07(0.04,0.09) | 3 | 276.94(146.25,444.43) | -0.01(-0.04,0.01) | 3 |
| Congo | 12.83(6.3,20.77) | -0.03(-0.06,0) | 3 | 279.24(143.2,446.87) | -0.2(-0.24,-0.14) | 3 |
| Democratic Republic of the Congo | 10.33(5.68,16.14) | 0.08(0.04,0.11) | 3 | 233.94(131.31,363.45) | 0.02(-0.02,0.07) | 3 |
| Equatorial Guinea | 12.33(5.47,20.98) | 0.55(0.45,0.64) | 2 | 265.22(131.89,442.98) | 0.27(0.19,0.38) | 4 |
| Gabon | 14.23(6.28,22.9) | 1.28(1.24,1.34) | 4 | 296.43(141.96,478.87) | 1.12(1.07,1.17) | 4 |
| Australasia |  |  |  |  |  |  |
| Australia | 1.99(1.13,2.83) | 0.25(-0.01,0.45) | 1 | 40.98(23.17,58.87) | -0.01(-0.15,0.1) | 1 |
| New Zealand | 2.2(1.25,3.17) | 1.04(0.82,1.27) | 1 | 49.81(29.25,71.96) | 0.63(0.45,0.81) | 1 |
| High-income Asia Pacific |  |  |  |  |  |  |
| Brunei Darussalam | 8.5(4.6,13.59) | -0.35(-0.47,-0.25) | 5 | 162.41(88.27,255.51) | -0.45(-0.54,-0.36) | 5 |
| Japan | 2.14(1.17,3.33) | -1.4(-1.49,-1.27) | 1 | 49.92(27.22,74.37) | -1.35(-1.44,-1.25) | 1 |
| Singapore | 1.73(0.91,2.76) | -1.68(-1.87,-1.51) | 1 | 37.4(20.18,58.43) | -1.83(-1.98,-1.68) | 1 |
| Republic of Korea | 2.4(1.31,3.61) | -1.87(-1.96,-1.78) | 1 | 40.63(22.17,61.13) | -2.09(-2.15,-2.02) | 1 |
| Tropical Latin America |  |  |  |  |  |  |
| Brazil | 5.13(3.2,7.01) | 0.08(-0.02,0.19) | 6 | 123.64(76.83,167.39) | -0.23(-0.32,-0.12) | 6 |
| Paraguay | 6.74(3.86,10.31) | 1.2(1.08,1.3) | 6 | 155.49(91.3,234.33) | 1.04(0.94,1.15) | 6 |
| Southeast Asia |  |  |  |  |  |  |
| Cambodia | 6.17(3.25,9.55) | 0.34(0.33,0.35) | 5 | 141.49(75.56,224.21) | 0.02(0.01,0.03) | 5 |
| Indonesia | 6.26(3.59,9.68) | 0.63(0.6,0.66) | 5 | 157.54(90,246.44) | 0.31(0.29,0.33) | 5 |
| Laos | 9.05(4.55,14.79) | -0.83(-0.84,-0.81) | 5 | 205.45(104.77,345.32) | -1.12(-1.13,-1.1) | 5 |
| Malaysia | 7.72(4.51,11.68) | 0.32(0.15,0.53) | 5 | 179.45(101.85,270.3) | 0.11(-0.02,0.31) | 5 |
| Maldives | 6.93(3.63,10.87) | -2.11(-2.2,-2) | 5 | 139.05(73.05,218.55) | -2.57(-2.66,-2.46) | 5 |
| Mauritius | 19.9(11.54,28.83) | 1.45(1.23,1.72) | 2 | 455.36(266.72,656.57) | 1.36(1.09,1.65) | 2 |
| Myanmar | 6.28(3.47,9.95) | -0.44(-0.46,-0.42) | 5 | 147.35(80.67,230.57) | -0.77(-0.79,-0.75) | 5 |
| Philippines | 8.82(4.57,13.55) | 0.54(0.44,0.67) | 5 | 209.55(108.16,319.57) | 0.84(0.77,0.9) | 5 |
| Sri Lanka | 5.65(2.92,9.55) | -1.24(-1.44,-1.06) | 5 | 130.73(67.47,216.57) | -0.96(-1.07,-0.83) | 5 |
| Seychelles | 11.39(6.18,17.49) | 1.14(0.94,1.33) | 5 | 247.09(134.24,375.95) | 0.7(0.55,0.83) | 5 |
| Thailand | 6.2(3.06,9.89) | 0.17(0.05,0.26) | 5 | 138.04(69.67,219.51) | 0.19(0.1,0.3) | 5 |
| Timor-Leste | 8.83(4.61,14.23) | 0.48(0.45,0.51) | 5 | 203.06(106.76,329.35) | 0.35(0.31,0.4) | 5 |
| Vietnam | 6.48(3.36,10.31) | 0.01(-0.01,0.02) | 5 | 130.32(67.5,207.13) | -0.3(-0.32,-0.28) |  |
| Western Sub-Saharan Africa |  |  |  |  |  |  |
| Benin | 8.85(4.69,13.64) | 0.22(0.19,0.24) | 3 | 183.07(98.11,283.26) | 0.14(0.11,0.17) | 3 |
| Burkina Faso | 11.17(6.27,16.68) | 0.53(0.48,0.57) | 3 | 227.32(129.83,345.29) | 0.39(0.37,0.42) | 3 |
| Cameroon | 10.93(5.84,17.97) | 0.03(-0.04,0.09) | 3 | 236.48(126.49,387.63) | 0.11(0.04,0.17) | 3 |
| Cabo Verde | 6.32(3.04,10) | 1.71(1.55,1.87) | 3 | 128.9(65.67,202.66) | 1.08(0.98,1.2) |  |
| Chad | 7.97(4.32,12.73) | 0.54(0.52,0.58) | 3 | 175.42(95.19,280.2) | 0.52(0.48,0.57) | 3 |
| Cote d'Ivoire | 8.68(4.73,13.54) | 0.22(0.18,0.25) | 3 | 190.73(103.55,295.94) | 0.19(0.16,0.22) | 3 |
| Gambia | 11.49(6.19,17.72) | 0.83(0.74,0.92) | 3 | 245.13(133.74,375.5) | 0.85(0.73,0.98) | 3 |
| Ghana | 9.12(4.87,14.4) | 1.28(1.25,1.3) | 3 | 186.47(98.99,296.1) | 1.1(1.07,1.12) | 3 |
| Guinea | 6.88(3.56,10.99) | 0.53(0.52,0.55) | 3 | 153.77(81.03,247.96) | 0.57(0.55,0.58) | 3 |
| Guinea-Bissau | 11.05(6.08,17.27) | -0.15(-0.17,-0.13) | 3 | 248.33(135.37,389.44) | -0.24(-0.25,-0.22) | 3 |
| Liberia | 11.8(6.15,18.91) | 0.3(0.25,0.35) | 3 | 252.55(136.54,397.17) | 0.31(0.26,0.36) | 3 |
| Mali | 9.03(4.84,13.63) | -0.08(-0.12,-0.04) | 3 | 186.41(101.65,283.69) | -0.15(-0.18,-0.13) | 3 |
| Mauritania | 11.23(5.83,18.14) | -0.02(-0.05,0.01) | 3 | 224.16(120.13,356.18) | -0.24(-0.29,-0.19) | 3 |
| Niger | 6.81(3.71,10.88) | -0.29(-0.31,-0.27) | 3 | 145.37(80.57,229.64) | -0.45(-0.48,-0.42) | 3 |
| Nigeria | 6.88(3.61,10.46) | 0.29(0.27,0.32) | 3 | 149.47(78.49,228.58) | 0.24(0.23,0.26) | 3 |
| Sao Tome and Principe | 14.34(7.17,23.15) | 0.65(0.58,0.7) | 2 | 286.11(148.15,462.27) | 0.58(0.52,0.65) | 3 |
| Senegal | 10.7(5.7,16.88) | 0.06(-0.02,0.14) | 3 | 216.71(118.33,351.27) | -0.13(-0.24,0.01) | 3 |
| Sierra Leone | 7.41(3.88,11.4) | -0.16(-0.18,-0.13) | 3 | 162.09(86.63,252.07) | -0.1(-0.12,-0.08) | 3 |
| Togo | 9.45(5.21,14.79) | 0.46(0.43,0.48) | 3 | 200.06(111.61,304.24) | 0.4(0.38,0.42) | 3 |
| Caribbean |  |  |  |  |  |  |
| Antigua and Barbuda | 10.44(6.17,14.69) | 1.3(1.07,1.49) | 3 | 223.01(137.61,308.72) | 0.95(0.75,1.11) | 3 |
| Bahamas | 7.97(4.75,11.69) | 1.49(1.31,1.63) | 6 | 199(121.43,289.41) | 1.29(1.14,1.42) | 6 |
| Barbados | 7.7(4.56,11.4) | 1.17(0.94,1.38) | 6 | 172.48(102.05,251.71) | 0.86(0.66,1.06) | 6 |
| Belize | 8.62(5.13,12.26) | 1.75(1.6,1.89) | 6 | 210.01(127.83,294.41) | 1.66(1.5,1.81) | 6 |
| Bermuda | 3.96(2.39,5.75) | 0.18(0.01,0.32) | 6 | 88.57(54.14,126.88) | 0.1(-0.06,0.23) | 6 |
| Cuba | 3.21(2,4.49) | 1.52(1.39,1.67) | 6 | 77.36(47.84,108.83) | 0.93(0.82,1.05) | 6 |
| Dominica | 8.1(4.73,12.29) | 1.18(1.16,1.22) | 6 | 186.49(110.81,277.17) | 1.26(1.23,1.28) | 6 |
| Dominican Republic | 4.77(2.57,7.43) | 0.63(0.54,0.74) | 6 | 118.64(65.06,182.26) | 0.69(0.61,0.77) | 6 |
| Grenada | 13.18(7.77,18.7) | 1.59(1.42,1.74) | 2 | 310.64(193.44,436.61) | 1.25(1.12,1.37) | 2 |
| Guyana | 13.45(8.04,20.35) | 1.7(1.49,1.88) | 2 | 341.02(204.36,508.19) | 1.71(1.5,1.86) | 2 |
| Haiti | 8.03(3.75,17.16) | 0.41(0.37,0.44) | 3 | 207.63(99.28,417.2) | 0.38(0.35,0.41) | 3 |
| Jamaica | 6.5(3.72,10.08) | 1.07(0.69,1.56) | 3 | 162.8(94.15,242.52) | 1.37(0.85,1.8) | 3 |
| Puerto Rico | 7.08(4.15,10.24) | -0.55(-0.75,-0.37) | 6 | 161.26(96.21,230.13) | -0.4(-0.68,-0.08) | 6 |
| Saint Kitts and Nevis | 15.3(9.36,21.83) | 0.72(0.58,0.87) | 2 | 331.11(207.58,466.65) | 0.37(0.25,0.51) | 2 |
| Saint Lucia | 9.93(6.01,14.2) | 0.32(0.23,0.42) | 3 | 230.43(142.82,321.56) | 0.42(0.32,0.53) | 3 |
| Saint Vincent and the Grenadines | 9.2(5.57,12.9) | 0.83(0.68,0.96) | 3 | 214.86(133.96,300.13) | 0.73(0.59,0.86) | 3 |
| Suriname | 9.12(5.18,13.88) | 1.11(0.97,1.25) | 3 | 232.15(134.97,343.97) | 1(0.87,1.13) | 3 |
| Trinidad and Tobago | 10.78(6.74,16.22) | 1.55(1.38,1.72) | 2 | 270.12(167.42,398.5) | 1.69(1.49,1.87) | 2 |
| United States Virgin Islands | 4.41(2.46,6.71) | -0.19(-0.28,-0.12) | 6 | 116.84(66.71,170.07) | -0.02(-0.1,0.04) | 6 |
